# The political economy of healthcare reform in China: negotiating public and private

**DOI:** 10.1186/2193-1801-2-448

**Published:** 2013-09-10

**Authors:** Arthur Daemmrich

**Affiliations:** Department of History and Philosophy of Medicine, University of Kansas Medical Center, 2025 Robinson Hall / MS 1025, 3901 Rainbow Boulevard, Kansas City, KS 66160 USA

**Keywords:** China, Healthcare reform, Clinical trials, Electronic medical records, Insurance, Pharmaceuticals

## Abstract

China’s healthcare system is experiencing significant growth from expanded government-backed insurance, greater public-sector spending on hospitals, and the introduction of private insurance and for-profit clinics. An incremental reform process has sought to develop market incentives for medical innovation and liberalize physician compensation and hospital finance while continuing to keep basic care affordable to a large population that pays for many components of care out-of-pocket. Additional changes presently under consideration by policymakers are likely to further restructure insurance and the delivery of care and will alter competitive dynamics in major healthcare industries, notably pharmaceuticals, medical devices, and diagnostic testing. This article describes the institutional history of China’s healthcare system and identifies dilemmas emerging as the country negotiates divisions between public and private in healthcare. Building on this analysis, the article considers opportunities for public-private partnerships and greater systems integration to reconcile otherwise incommensurable approaches to rewarding innovation and improving access. The article concludes with observations on the public function of health insurance and its significance to further development of China’s healthcare system.

## Introduction

Healthcare is arguably the most complex of the institutional systems with which people interact on a regular basis. In every country, healthcare systems combine to differing degrees public state administration and private market economies for insurance and the provision of care. Debates about the superiority of predominantly public or private systems have a strong ideological component, and international comparisons are made difficult by the reality of subtly different mixes of public and private in each country and the shifts induced over time by frequent reforms (Saltman 2003; Weintraub 1997). Health systems nonetheless exhibit core identities based on the division of power between public and private that are open to negotiation at certain historical moments but otherwise closed off to significant reform possibilities (Rosenberg 2007). This article finds that that the underlying identity and institutional design of China’s health system is presently in flux. As policymakers negotiate what counts as “public” and the role of “private” in health insurance and the delivery of care, they also are encountering a dilemma between supporting profit-seeking industries that offer the potential for new medical products and services but want free-market pricing, and public access to low-cost care that requires redistributive policies and price controls to function efficiently.

A decade of reforms to China’s healthcare system has significantly broadened government-backed insurance coverage and the availability of basic care. Industry analysts predict healthcare spending in China to grow further to $1 trillion by 2020, which would triple 2010 levels and then comprise an estimated 7 percent of GDP (Le Deu et al. 2012). Policy discussions concerning where to direct additional public spending and the degree of private enterprise that is desirable in China’s future healthcare system are underway following a merger in early 2013 of the Ministry of Health and the National Population and Family Planning Commission to create the National Health and Family Planning Commission (H&FPC). While many analysts expect further liberalization of insurance and hospitals, greater government spending on health also will bring increased regulatory oversight and additional demands for access to care and the protection of public interests.

Reflecting on these issues, the deputy director of the powerful National Development and Reform Commission emphasized “three basics as a guiding ideology” at a press conference during the 12th National People’s Congress in March, 2013. The “basics” include universal basic health services, a basic principle of grass-roots orientation to care, and a basic path to further reform through evolutionary developments.^a^ Other ministers speaking about the healthcare system discussed significant additional spending on insurance and care in order to reduce hospital reliance on drug sales, raise physician salaries, and shorten waiting times for patients.

Reforms under discussion may allow greater sale of private insurance, privatize additional public hospitals, and license new for-profit clinics. However, pressures also will increase for the government to instead develop a single-payer national insurance scheme and mandate the use of inexpensive generics to bring down prescription drug spending. China’s public has benefitted from a significant increase in lifespan and an 11.5 percent decrease in infant mortality between 2000 and 2010 (OECD 2013). Tensions nevertheless are apparent concerning access to care as hospitals build exclusive care suites, doctors have incentives to prescribe imported brand-name drugs while minimizing time spent with patients, and people crowd into top-ranked urban hospitals. As a result, reform to China’s healthcare system is an exercise in drawing boundaries between public and private.

This article analyzes the historical and institutional trajectory of the Chinese healthcare system, identifies dilemmas confronting healthcare reforms, and considers initiatives underway in pharmaceutical research and the development of electronic medical record systems. Particular focus is put on shifts between public and private provision of insurance and medical care. I argue that the Chinese healthcare system is at an inflection point between greater centralization and liberalization. Decisions taken by policymakers over the next several years will determine the future of private insurance, the viability of new business models for private hospitals, and the competitive dynamics for domestic and multinational pharmaceutical, device, and diagnostics firms.

The analysis developed here is based on diverse quantitative and qualitative sources. The author conducted observations at four hospitals and specialty clinics in Shanghai and Xi’an, and carried out interviews with experts at insurance, pharmaceutical, medical device, diagnostics, and information technology (IT) companies. The article also draws upon published data and official reports from the former Ministry of Health and other government ministries, interviews and speeches by government officials, commercial databases accessed through university subscription, and existing scholarship in health economics and policy.

This article adopts a historical perspective on institutional change while also drawing on concepts from health economics and political science concerning public, private, and hybrid institutions. A longstanding body of research has examined markets as optimal for the production and exchange of private goods, while government is needed to generate public goods and regulate areas subject to market failure (Samuelson 1954; Breyer 1982). But as the economist Kenneth Arrow (1963) suggested, the provision of medical care is complicated since it exhibits dimensions of public goods (e.g., preventing disease contagion or keeping people productive) and private goods (e.g., seeing a physician or purchasing prescription drugs). As a consequence, nearly every country’s healthcare system has a mix of public and private insurance, public and private delivery of care, and public and private support for the development of new medicines and treatments (Johnson and Stoskopf 2010).

Divisions of authority, responsibility, and allowance of profit seeking that develop over time are revealing of the underlying identity of a healthcare system. China offers a rare example of a country that oscillated over a short period of time between a public-private mix in the pre-WWII period, to fully socialized healthcare from 1949 through the late 1970s, to a mixed system at present (Barber and Yao 2011). This history makes China an especially interesting country in which to examine shifts between public and private in healthcare and the tradeoffs that arise even during an expansionary reform process.

The article proceeds in three major sections. The first develops an institutional history of healthcare in China across traditional Chinese medicine, public health under Mao Zedong, and health policy during the most recent three decades of market-oriented reforms. The second section identifies challenges for the current system based on China’s three basic public insurance schemes, competing forces affecting hospitals, tensions in the medical profession, incentives for over-use of profit-generating care, and changing demographics and disease dynamics. Third, the article considers China’s potential to take a global lead for clinical trials of new medicines and the development of electronic medical records through greater public-private partnerships. Throughout, I emphasize continuities in healthcare institutions as well as areas where policymakers are breaking with the past by facilitating an increased role for the private sector.

### Institutional history of healthcare in China

Policymakers in China presently face a daunting set of challenges in the reform and expansion of the health system. Large urban hospitals (tier-II and tier-III hospitals in China’s domestic ranking system, under which tier-III are the best) earn revenue from markups on diagnostics and drug sales, leading direct financial incentives for doctors to over-prescribe brand-name drugs and diagnostic tests (Ramesh et al. 2013). Medical infrastructure varies tremendously among regions and capital spending lags other sectors. The insurance system is underdeveloped and consumer information necessary for effective risk management and risk pricing is lacking. The quality of medicines varies widely and counterfeits of prescription drugs and traditional Chinese medicines are widespread. Thanks to the interconnected nature of the healthcare system, any regulatory changes will have repercussions on incentives for physicians, hospitals, insurers, and pharmaceutical and medical device firms. Understanding the historical development of China’s healthcare institutions is a first step to analyzing contemporary negotiations on the balance between public and private.

#### Traditional Chinese medicine

With a recorded history dating over 3,000 years, traditional Chinese medicine (TCM) encompasses a range of practices including herbal medicine, acupuncture, therapeutic massage, movement and exercise, and dietary therapy (Sivin 1987; Liao 2011). TCM has sustained a strong institutional basis in contemporary China, with large medical universities, specialized hospitals, purpose-built divisions within general hospitals, and private clinics around the country. Approximately 2,000 TCM companies produce plant-based and synthetic therapeutics in forms ranging from teas to pills. Systematic research efforts are underway to characterize TCM therapies using modern analytical chemistry instrumentation and to run formal clinical trials of TCM drugs (Li et al. 2008).

Historically, TCM practitioners were paid at the point of care, although some wealthier Chinese paid a retainer to their doctor to keep them healthy. If they became ill, the doctor returned part of the fee. In contemporary hospitals and TCM clinics, patients pay on a fee-for-service basis. Thanks to a concerted government effort to sustain TCM alongside Western medicine, some physicians employ both therapeutic approaches. In most hospitals, however, patients choose whether to be seen by a practitioner of TCM or of Western medicine. Physicians of either therapeutic direction routinely prescribe a combination of drugs as in the West and herbals or teas from the TCM *materia medica*. Hospital pharmacies stock TCM treatments alongside small molecule drugs. In addition, specialized TCM clinics can be found across the country at which people can build long-term relationships with their doctor, in contrast to crowded hospitals where patients typically must see the next available physician (Xu and Yang 2009).

#### “Barefoot Doctors” and public health

At the inauguration of the People’s Republic of China in 1949, the country had only 40,000 doctors to care for a population of nearly 540 million. Despite a low urbanization rate, most physicians were concentrated in cities. Infectious and parasitic diseases were endemic and epidemics broke out with frequency in the countryside. Mao considered the communist movement to have originated in the peasant class and the party consequently made rural healthcare one of its top priorities.

New care providers were trained in three- to six-month crash courses starting in the early 1950s and sent to open rural clinics. Most also were expected to work in the fields and received additional medical training during winter months. By 1957, over 200,000 army veteran doctors and nurses, quickly-trained physicians, and TCM doctors were providing frontline care across China, notably by treating wounds, providing antibiotics, assisting in childbirth, vaccinating children, and advising on basic hygiene (Zhang and Unschuld 2008). The mix of care providers gained significant institutional support after a 1965 speech in which Mao stated, “In medical and health work, put the stress on the rural areas” (Wen 1974). Now named “barefoot doctors,” the frontline practitioners also gained international recognition. Public health measures such as building public latrines and draining swamps sharply reduced infectious disease. One widely acclaimed success was the near elimination of the parasitic disease schistosomiasis.

Between the mid-1960s and mid-1980s, healthcare in China was structured into a three-tiered system termed the Cooperative Medical Scheme. The first tier consisted of over one million barefoot doctors by the mid-1970s. More complex or emergency cases were directed to the second tier of commune-based health clinics funded by local residents. A commune health clinic typically had 10 to 30 beds and one or two trained junior doctors. When more serious care was needed, patients were transferred to larger county or city hospitals; by the mid-1970s, each county in China operated a general hospital (Wen 1974). Communes paid fees for care out of pooled funds.

Urban residents received care directly at hospitals, typically seeing the first available physician after registering. The Cultural Revolution undermined medical services in the cities, especially when some senior physicians were branded as bourgeois counter-revolutionaries and many younger doctors were sent to rural areas. Medical education experienced significant setbacks when universities were closed for long periods. Broadly, health policy from the 1950s through the 1980s was characterized by a focus on meeting the basic health needs of peasants, soldiers, and workers. Healthcare was seen as an integral component of the communist system and important to the development of a collective Maoist Chinese identity. Private firms and market incentives were eliminated throughout the system, from hospitals to drug manufacturing (Yang 2010).

#### Market reforms and healthcare policy

Beginning in 1978, a gradual and sequential set of reforms encouraged the formation of township and village enterprises, created special economic zones for export-oriented businesses, and partially liberalized the financial sector. Subsequent economic reforms in the 1980s ended the commune system and reduced foreign investment and trade restrictions (Meessen and Bloom 2007). In 1985, the ministry of health phased out support for barefoot doctors (Zhang and Unschuld 2008). Those able to pass qualifying examinations were now termed “rural doctors”, while others were re-categorized as health workers or medical aides. Rural Chinese began to pay for care out of pocket, as was already common in cities. With policymakers focused on economic development and managing the introduction of market capitalism, healthcare was largely ignored and the Cooperative Medical Scheme collapsed (Zhang 1999; Liu et al. 2006). Between 1980 and 2000, out-of-pocket spending by individuals thus increased from 21 percent to 60 percent of all health expenditures (see Table 1). The government’s share, by contrast, shrank from over 36 percent to 14 percent (Ministry of Health 2012a). Nevertheless, hospitals remained dominated by state ownership and government control. Few private clinics were opened.

**Table 1:** **Healthcare expenditures and sources of healthcare funding in China**

Year	Healthcare spending (% GDP)	Government (%)^a^	Social security insurance (%)^a^	Out-of-pocket payments (%)^a^
1980	3.15	36.2	42.6	21.2
1985	3.09	38.6	33.0	28.5
1990	4.00	25.1	39.2	35.7
1995	3.54	18.0	35.6	46.4
2000	4.62	15.5	25.6	59.0
2005	4.68	17.9	29.9	52.2
2010	5.01	28.6	35.9	35.5

^a^Percentage of total healthcare spending.

Source: P.R. China. 2012. *Health Statistical Digest*. Beijing: P.R. China.

At the same time, rapid economic growth in the coastal areas and migration to urban centers meant that China shifted from 19 percent of the population living in cities in 1980 to over 50 percent by 2011 (Meng 2010; China Daily 2012). Physicians and trained medical personnel likewise moved to cities; in the space of fifteen years, township health centers across China lost nearly all of their qualified doctors (Gong and Wilkes 1997). As the economy grew at double-digit rates, a middle class emerged. However, few employers provided health insurance and only expatriates had access to private insurance. Even in cities, health insurance became rare, with a decline in the number of people covered during the 1990s that eventually bottomed out at 40 percent (Liu et al. 2000).

Thus in the 1980s and 1990s, the Chinese principally self-insured. When ill, they drew upon savings, family funds, and friends and co-workers to pay medical expenses. As a result, the Ministry of Health in 2000 warned that nearly one-quarter of new cases of poverty could be attributed to medical expenses (Meng and Hu 2000). A follow-up study by health economists found a 44 percent increase in the number of rural households living in poverty as a result of out-of-pocket medical spending (Liu et al. 2003). According to a national survey conducted in 2003, nearly 50 percent of Chinese reporting an illness did not seek outpatient care and 30 percent of those advised by a physician to be hospitalized did not do so; in both cases, the majority cited financial concerns as the primary reason (Yip and Mahal 2008).

System integration also declined significantly, and patients fell into gaps between different components of the health system. For basic care, people had to wait in long queues at hospitals, with no possibility for scheduled appointments and little privacy during a brief physician consultation. Since patients did not have a regular primary care physician or clinic, they had to maintain and carry with them personal medical records. A lack of coordination among health facilities also contributed to a rise of communicable diseases in China in the 1990s and early 2000s (Liu 2004; Tang and Squire 2005).

Healthcare in this period was seen as a consumption activity rather than a fundamental right of the people or a responsibility of the state as part of its goals for economic growth. Conceptually, health shifted from a public good to the private responsibility of each individual. Institutionally, most healthcare services remained public, with government-set prices for doctor visits, surgery, and other care. But the provision of medicines, diagnostic tests, surgical implants, and specialized care shifted to a free-market model.

### Dilemmas for contemporary health system reform

#### Insurance

Starting in the mid-2000s, the Chinese government began to increase spending on healthcare, mostly to expand insurance coverage but also for biomedical infrastructure of hospitals and research facilities (Barber and Yao 2011). A series of pilot projects begun in 300 rural counties in 2003 subsequently evolved into the New Cooperative Medical Scheme (NCMS) on the national level (Zhang et al. 2010). Restricted to rural participants, NCMS offers very basic subsidized insurance to households. At its origin, a typical insured member paid an annual contribution of 10 RMB ($1.60)^b^, with additional contributions by the central and local governments of 20 RMB each; premiums and coverage subsequently rose, though only modestly. Administration of NCMS and coverage decisions are the responsibility of local governments, who manage budgets by setting reimbursement limits. At present, coverage is limited to inpatient care and annual ceilings per patient are used to control expenditures. In 2012, the government set a common formula for coverage limits of eight times average annual farm income, but not less than 60,000 RMB ($9,600). NCMS operates on a local basis, and deductibles and copayments typically increase for treatment away from home (Brown and Theoharides 2009).

Although NCMS is voluntary, it was designed from the start with sufficient scale to allow for risk pooling. The central government encouraged provinces to actively build membership through a baseline requirement of 80 percent participation for receipt of subsidies (You and Kobayashi 2009). While payment models such as capitation were used on an experimental basis in the early 2000s, fee-for-service emerged as the dominant approach by the end of the decade. Studies of NCMS have found that the utilization of services rose, but its implementation also increased out-of-pocket spending since insurance did not cover chronic disease care or most branded prescription drugs (Yip and Hsiao 2009; Wagstaff et al. 2007).

For working urban residents, health insurance has been available since 1998 through the Urban Employee Basic Medical Insurance (UEBMI) program, which covers employees in private and state-owned enterprises, government, social organizations, and non-profits. Employees of state-owned enterprises and their dependents were previously covered under a government employee insurance scheme. In 2007, the government ran pilot programs in 79 cities to test coverage of non-working urban residents, including family members of people covered by UEBMI. This new Urban Residents Basic Medical Insurance (URBMI) was expanded nationally the following year (Liu and Zhao 2012). Some 420 million non-salaried elderly, unemployed, and children in cities now obtain inexpensive coverage through URMBI. Premiums range across the country, but are low by international standards. Government subsidies in 2011 were 200 RMB per person ($32).

Similar to the rural program, URBMI operates at the city level following broad central government guidelines and participation is voluntary. As with NCMS, utilization rates and out-of-pocket spending increased following implementation of the urban insurance schemes, since many prescription drugs were not covered and URMBI pays for only 45 percent of inpatient medical costs (Lin et al. 2000).

As part of a package of fiscal stimulus measures in 2009, the government announced 850 billion RMB ($136 billion) in new health sector spending, spread among the urban and rural insurance schemes (National Development and Reform Commission 2009). For NCMS, premium subsidies were raised to 120 RMB ($19.20). Urban insurance programs also benefitted, as UEBMI was extended to over 180 million enrollees, and URBMI became comprehensive in reach. Annual ceilings were raised to six times the local average wage for UEBMI and six times the local per capita disposable income for URBMI. Although direct spending on rural and urban insurance was the most visible component of the 2009 program, plans also were announced for new hospital construction, renovations to existing facilities, and policy changes to create an essential drug list and initiate public hospital finance reform.

When announcing the 2009 healthcare package, the Central Committee and State Council took note of mounting tensions:

Health care undertakings are developing unevenly between urban and rural areas and among different regions; resource allocation is unreasonable; the work of public health as well as rural and community health care is comparatively weak; the medical insurance system is incomplete; pharmaceutical production and circulation is not well regulated; the hospital managerial system and operational mechanism are imperfect; government investment in health is insufficient; medical costs are soaring individual burden is too heavy, and therefore, the people’s reaction is very strong (National Development and Reform Commission 2009).

Government spending and incentives for participation nonetheless expanded basic coverage from an estimated low point of 40 percent of urban residents and only 4 percent of rural dwellers in the late 1990s to 95 percent overall by 2012 (Meng and Tang 2010; People’s Republic of China 2012a). Patients across the country now can see a physician at little or no direct cost, either at an urban public hospital or at a rural clinic. But diagnostic tests and treatments ranging from prescription drugs to surgery still require advance out-of-pocket payments, and only a small subset of the population is covered by employer-sponsored or other supplemental insurance.

Seeking to build on its success at expanding basic coverage, in 2012 the Ministry of Health announced a special fund for 125 billion RMB ($20 billion) in new healthcare spending alongside a proposal to expand insurance plans by offering catastrophic disease coverage nationwide by 2020. Specifics remain to be developed, but the government has proposed covering treatment costs above an upper limit of 50,000 RMB ($8,000), either completely or on a sliding scale. However, as the H&FPC plans additional reforms, institutionalized behaviors among the key players in the healthcare system are challenging the balance it seeks to strike between public and private in insurance and the delivery of care.

#### Hospitals

With few exceptions, hospitals in China are built, owned, and operated by public authorities. They receive national and provincial or city government funding based on metrics such as the number of beds filled on a monthly or annual basis and staff size relative to the number of patients treated and discharged. At the same time, the government limits hospital revenue through the “Yellow Book”, a price list for thousands of medical procedures and services, many set at or even below their direct cost (Liu and Mills 2005). With a 15 percent markup permitted on prescription drugs and diagnostic testing, hospitals earn up to 50 percent of their revenue and in many cases 90 percent of profits from just these two areas.

Hospitals serve multiple functions in China’s healthcare system in the absence of private practices or specialty clinics. They have strong incentivizes to maximize the number of patients seen by doctors, make heavy use of diagnostic and scanning equipment, and push branded prescription drugs. Since patients typically fill prescriptions on-site (80 percent of retail pharmaceutical sales take place in hospital pharmacies), hospitals capture the difference between wholesale and retail prices. Furthermore, studies of health spending in China suggest that hospitals regularly receive kickbacks from drug companies and medical suppliers (Yip et al. 2010). As a consequence, 75 percent of patients suffering from common colds and 79 percent of all hospitalized patients are prescribed antibiotics, well over double the global average (Yip and Hsiao 2008). A similar pattern has been identified for hormones (notably corticosteroids) and injected and infused antibiotics, which are prescribed at rates triple those of other middle-income countries (Li et al. 2012; Reynolds and McKee 2011).

Critics within China lament the high-speed, high-throughput approach to patient care common across the country. At the same time, hospitals achieve remarkably fast service at low cost. Despite only 1.5 practicing physicians per 1,000 population (contrasted with 2.4 per 1,000 in the United States and 3.5 per 1,000 in Germany) China’s hospitals handled 2.3 billion outpatient visits and 108 million inpatients in 2011 (People’s Republic of China 2012a). Still, hospitals are criticized for profiteering from excess care for those able to pay, while ignoring or under-treating poorer patients. Government leaders including former Health Minister Zhu Chen have commented openly on the need to improve hospital efficiency and deal with public concerns regarding “poor access and high fees” (Fei 2012). Hospitals nevertheless are public in ownership and financing, even as a small number of specialty clinics and VIP treatment centers with a maximum of 50 beds have been licensed in wealthier cities.

One model for private clinics involves their construction adjacent or in close proximity to existing top-tier urban hospitals. Clinics such as those operated by Parkway Health, a Singaporean company, offer better facilities and advance scheduling of doctor visits and surgical procedures. Private clinics employ the same physicians that work in public hospitals, but with significantly better compensation. Wealthier patients enjoy a better experience of high quality facilities and minimal wait times even as some of the population pressure on the public system is reduced. However, this initially modest degree of privatization could evolve to better care for the wealthy and greater social fragmentation. Supporters of private care delivery argue that physicians will continue to work in public hospitals for career advancement and prestige even as they offset poor compensation from public hospitals.

#### Medical profession

Employed principally by hospitals, physicians in China are in the process of building professional associations and establishing a corpus of medical ethics and codes of practice beyond the communist party mandate to “serve the people wholeheartedly” (Yang 2010). Base salaries are notoriously low, with a national average of only 4,200 RMB monthly ($672) (Chinese Medical Doctor Association 2011). Even in top-tier cities, such as Shanghai, monthly pay starts at 5,000 RMB ($800) for young doctors and rises to just 10,000 RMB ($1,600) for experienced physicians. But doctors typically also receive a significant yearly bonus that adds 40 percent or more to their salaries. Bonuses are based on the hospital’s overall revenue or the quantity of services a physician provides, including admissions, medical procedures, tests, and prescriptions (Yip et al. 2010). In a study critical of the practice, public health scholars noted that admissions had doubled and the frequency of operations tripled at hospitals that implemented revenue-related bonus systems (Liu and Mills 2005). Overall, concerns have been raised that Chinese doctors are implicated in a system with financial incentives to provide unnecessary care. Physicians and ethicists warn that medical judgment is being distorted (Chen 2007).

Beyond salaries and bonuses, physicians earn significant additional compensation from informal payments by patients, typically in the form of a red envelope (*hóng bāo*) containing cash. A regulation proposed by the Ministry of Health in August 2012 would require doctors and patients to sign an agreement in which the doctor vows not to ask for money and the patient vows not to try to hand over an envelope (Qingyun 2012). Since *hóng bāo* is transferred in private and the practice is deeply engrained in a Chinese gift culture that fosters relational ties, it is unlikely that the rule by itself will change the practice.

Medical education also is a topic of reform discussions. Students of clinical medicine enroll directly from secondary school for medical degrees that require three (diploma), five (bachelors), six (bachelors), seven (masters), or eight (full M.D.) years of education. Specialists have another two years of training (Xu et al. 2010). As part of the 2009 reforms, the government reversed previous plans to phase out 3-year programs and instead refocused them on training for rural primary care. Even as the country struggles with a shortage of primary care physicians, concerns have been raised that medical schools admit too many students, weakening the quality of education and generating a shortage of clinical internship positions (Ba 2007). The combination of openings for students but low compensation for physicians has produced a peculiar employment dynamic in China. Until the late 1990s, nearly every new doctor went to work as practicing physicians. But in the decade between 2000 and 2010, the majority of medical graduates found employment outside of general medicine, often for pharmaceutical or medical device firms (People’s Republic of China 2012b).

#### Strained doctor-patient relationships

As a result of the incentive for hospitals to speed patients through consultations and sell them medicines, doctor-patient relations are severely strained. Patients resent a lack of privacy in hospitals, complain on the Internet about better care for well-connected people, and perceive doctors as arrogant and uncaring. Tensions have mounted with a spate of attacks on medical personnel by patients and their families, averaging one per month in 2010 and 2011 (Jiang 2011; Yingqi 2011).^c^

For their part, physicians express severe stress concerning workload and poor pay. Doctors commonly see twenty or more patients per hour and in some clinics, patients pile into doctors’ offices while jostling for a position. Noting the challenges for physicians, the vice minister of health, Huang Jiefu, stated, “How could a doctor maintain a good attitude for all day long, with no time to take a rest, to drink, and to go to the toilet?” (Fei 2012). According to a poll conducted by the Chinese Medical Doctor Association in 2011, 95 percent of physicians are dissatisfied with wages, which are just 20 percent above the national average for all workers (Chinese Medical Doctor Association 2011). Broadly, physicians are finding it challenging to develop professional autonomy relative to hospitals that employ them or a government that sets wages and other conditions of employment.

#### Biopharmaceuticals, devices, and diagnostics

Spending on prescription drugs in China took off in the mid-1990s, expanding since at an average annual rate of 20 percent (World Bank 2010). Total pharmaceutical sales were 432 billion RMB ($69 billion) in 2011 and are projected to grow to 521 billion RMB ($83 billion) in 2012 and 625 billion RMB ($100 billion) in 2013 (Business Monitor International 2012). Alongside tremendous sales growth, the market shifted to imported and joint-venture branded drugs, with TCM and generics in relative decline. Domestic products thus slid to 60 percent of drug sales in 2010 (World Bank 2010).

The potential size of China’s healthcare market has contributed to multinational firms making significant long-term investments in the country. Projects that tap into China’s world-leading graduation of Ph.D. chemists and biomedical scientists are multiplying; according to a McKinsey Consulting report, “13 of the top 20 pharmacos [sic] have established R&D centers in China, and several have announced major manufacturing investments” (Le Deu et al. 2012). Venture capital also has flowed into China, with 70 VC-backed biotechnology companies present in 2012, more than double that of any other emerging market (Chakma et al. 2013). Drug marketing, a hallmark of private-sector healthcare, has grown exponentially. The total number of pharmaceutical sales representatives in China outnumbered those in the United States in 2012 (Le Deu et al. 2012).

Physicians and public health scholars have criticized incentives for hospitals in China to push high-technology services and expensive imported drugs to underwrite profit margins (Blumenthal and Hsiao 2005). However, world-class diagnostics and branded prescription drugs also enjoy market pull in China from a growing middle class and wealthy urban populations. An initiative in the early 2000s that sought to reduce drug prices in China through direct government controls produced unintended consequences of drug shortages; patients then were then switched to other costly drugs or less effective alternatives (Meng et al. 2005; Chen and Schweitzer 2008). The government subsequently supported free pricing of drugs even though H&FPC has the mandate to develop an essential drugs list as part of a plan to ensure the availability of low-cost treatment choices nationwide.

Medical device firms also are enjoying growth from market dynamics that encourage hospitals to acquire new technologies and use them extensively. The device market was estimated at 146 billion RMB ($23 billion) in 2012 (Business Monitor International 2012). In some notable cases, early entrants have developed new imaging devices and diagnostic tests in China for Chinese patients (Johnson & Johnson 2011). Yet foreign firms often find it challenging to understand the hospital procurement system for devices and diagnostics. Consequently, some firms are switching from a strategy of globally branded product development to partnerships with local Chinese companies.

China’s domestic biopharmaceutical, device, and TCM firms also are experiencing rapid growth. Historically, provincial governments promoted local firms, leading to a patchwork of over 5,000 small-scale generics producers. Industry concentration is low, with the top 100 firms only accounting for one-third of national drug sales (Sun et al. 2008). While some consolidation has taken place in the past five years, new research-oriented companies also are being founded at a rapid rate, often with scientific collaborations and board members from the United States and European countries. Domestic firms thus occupy a complicated position, serving local public interests of providing employment and supplying low-cost therapies even as policymakers ask them to generate profits like other private firms.

Overall, China’s prescription drug policies are at an inflection point. The country is home to 20 percent of the world’s population, but consumed only seven percent of total global drug expenditures and was a host to only five percent of multi-sited clinical trials underway in 2012.^d^ If firms invest in research and development on par with the country’s rising international economic role, medical industries will experience a major global shift. However, unrestrained drug pricing by private firms is subject to criticism for the opportunity costs imposed on other parts of the healthcare system. With tighter price regulation, China could broaden access. However, price controls or prescription drug lists restricted to domestic generics would undermine incentives for the pharmaceutical industry to grow through research investments and new product development.

#### Demographics and disease

The healthcare system drew international scrutiny in 2003 when an outbreak of Severe Acute Respiratory Syndrome (SARS) in southern China spread to infect individuals in 37 countries. SARS eventually infected only 8,500 people worldwide with just 916 recorded fatalities (Kleinman and Watson 2006). To senior Chinese health officials, however, delays in identifying SARS and taking action were an embarrassment. Significant investment in public health and disease monitoring followed. The disease continues to be invoked regularly by officials advocating for a greater role for public health in China’s healthcare system (Chan et al. 2010).

Changes in lifestyle, diet, and the environment that are accompanying China’s fast industrialization and economic growth also are producing a national epidemiological transition. Chronic diseases have become a significant burden across the country, even as infectious diseases including tuberculosis and viral hepatitis are still prevalent (Yang et al. 2008). A 2010 study found that nearly 10 percent of the population has type-2 diabetes while another 15 percent exhibit pre-diabetic blood sugar levels (Yang et al. 2010). Other ailments associated with wealthier Western nations, including lung and breast cancer, obesity, hypertension, and cardiovascular disease are rising in incidence (Normile 2010). According to a 2005 World Health Organization study, China’s economy is projected to lose $560 billion by 2015 due to premature deaths from chronic diseases (World Health Organization 2005).

China’s epidemiological transition also poses a challenge to physicians with little experience treating diseases long prevalent in the West. In the case of cancer, for example, Chinese physicians often continue to prescribe aggressive drug treatments and initiate painful surgical procedures after evidence suggests a shift to palliative care would be preferable (Want et al. 2004). Understanding of co-morbidities such as depression is low among doctors treating cancer and other life-threatening illnesses. In addition to parents overfeeding children, a behavior ingrained from periods of extreme scarcity, there is some evidence that elderly Chinese who lived through famines in the late 1950s and early 1960s are at an increased risk of developing hyperglycemia and type-2 diabetes (Li et al. 2010). But physicians find it uncomfortable to recommend that parents feed their children less or that adults reduce their caloric intake.

Demographic factors also will raise considerable challenges for China’s healthcare system in the future. The population aged sixty and older is projected to increase from 13 percent in 2012 to 34 percent in 2050. Overall, thanks to increasing life expectancies and the one-child rule, China is likely to age more rapidly than any country in history (United Nations 2011). Sustainable plans for public health spending will have to account for a smaller future working population.

#### Identity and goals

Reporting on its progress at the Eleventh National People’s Congress in March 2011, the National Development and Reform Commission described basic insurance coverage of 1.3 billion residents and celebrated: “Smooth progress was made in developing the medical and health service system” (National Development and Reform Commission 2011b). An ambitious plan is being followed:

By 2020, the basic health care system covering urban and rural residents shall have been fundamentally established. We shall have set up, across the country, a fairly complete public health service system and health care service system … a secured and relatively well regulated pharmaceutical supply system, a comparatively sound health care institution management and operational system … the multi-layer demands of the people for health care services shall be met preliminarily, and the health level of the people shall be further enhanced (National Development and Reform Commission 2009).

The Chinese healthcare system at present is struggling with an identity split. On the one hand, the system seeks to deliver low-cost care to the world’s largest population while also upgrading technologies, expertise, and the quality of care through market incentives. On the other hand, the system is seeking to maintain a balance between central regulatory oversight and provincial and local management of publicly owned and financed care delivery.

With a high degree of out-of-pocket spending on prescription drugs, but price controls on inpatient and outpatient care, China’s distribution of spending is peculiar in international comparison (see Table 2). Compared with developed countries, total healthcare expenditures per person in China are low, only four percent of those in the United States and 10 percent of the OECD average. The structure of healthcare expenditure varies internationally, but China stands out for prescription drugs taking up over 40 percent of all spending (compared to approximately 12 percent in other countries), while hospitalizations and outpatient care are far lower. As overall health spending in China rises, the mix of private spending on drugs and public spending on hospitals will undergo a shift. Present negotiations over public and private, and possible mixed-mandate public-private corporations, will determine whether the country aligns to other developed nations or pursues a unique path.

**Table 2:** **International healthcare expenditures (per capita, $US, PPP)**

	Total	Hospital (inpatient)	Outpatient care	Prescription drugs	Administration
China	352	85	82	146	4
France	3,835	1,357	1,082	626	280
Germany	4,187	1,245	1,279	627	233
Japan	2,979	1,431	810	556	50
United States	7,910	2,634	2,852	947	570
OECD Average	3,392	1,213	954	487	136

Data are from 2010, except Japan from 2009. Subcategories do not sum to total because some services were excluded, such as long-term care and nursing.

Sources: Adapted from P.R. China, Ministry of Health. 2012. *Health Yearbook 2011*. Beijing: MoH; OECD iLibrary. 2013. OECD Health Statistics, http://www.oecd-ilibrary.org; World Health Organization 2013. Global Health Observatory Data Repository, http://www.apps.who.int/ghodata; accessed March 2013.

### Opportunities in public-private collaborations

In China’s 12th five year plan, approved in March 2011, Communist Party leaders identified biomedicine as one of seven sectors targeted for growth and grouped it with energy and technology as Strategic Emerging Industries (SEIs) (National Development and Reform Commission 2011a). Together, the SEIs are expected to contribute 15 percent of the country’s GDP by 2020. But growth in healthcare sub-sectors, especially biopharmaceuticals, medical devices, and diagnostics, could impose opportunity costs on other spending priorities.

Health systems around the world suffer from fragmentation between components of care and locations for access to care. Reform efforts that target greater system integration commonly encounter a “trilemma” of difficult choices between access to health services, outcome quality, and affordability (Bohmer 2009). China has a unique opportunity to overcome this trilemma thanks to its large population, two decades of sustained economic expansion, and state capacity to undertake long-term planning. In particular, current decisions to accelerate spending on healthcare infrastructure and care come at an opportune moment for the integration of information technology, biomedical science, and medical technologies.

Even as roles and boundaries of public and private are determined for China’s healthcare system, opportunities also are emerging for novel institutional forms. For example, public-private partnerships (PPPs) involve a negotiated division of resources, responsibilities, and rewards between governments and private sector firms (Nishtar 2004). In theory, PPPs can deliver public goods more efficiently and at a lower cost than either governments acting without profit incentives or private firms operating without government-based authority and legitimacy (Valente and Crane 2010). PPPs have been criticized as giveaways to multinationals that put a market price on public goods (Rosenau 1999). Nevertheless, they may offer China a route to development of clinical trials and electronic medical records that would benefit the country through better patient care and outcomes as well as increased exports of high-value healthcare products and services.

#### Clinical trials in china

Analysts have warned in recent years that declining success in pharmaceutical research and cost-cutting measures in developed countries are reducing incentives for firms to invest in drug discovery (Pammolli et al. 2011). At the same time, tremendous unmet medical needs are present in aging populations. Countries worldwide are struggling to treat rising numbers of patients with diabetes, rheumatoid arthritis, Parkinson’s, and Alzheimer’s diseases. New treatments are likely to require health and genetic data from large patient populations, in addition to big clinical trials. Combining a growing scientific workforce and a large patient population, China has the potential to build domestic firms that develop and market drugs globally and build clinical trials into a profitable international service business.

Drug development, including pre-clinical discovery, the modification of compounds to make them safer and more effective, and clinical testing all remain underdeveloped in China. Seeking to overcome technical barriers, government-sponsored projects are underway to develop standards for pharmacokinetics, pharmacodynamics, and other aspects of drug formulation. But policy concerns about multinationals exploiting Chinese patients have led to a process requiring 10 to 18 months to authorize new trials. Likewise, accreditation of clinical trial sites in China requires national and local government reviews taking six months or more (Le Deu et al. 2012).

Rather than serve solely as an outsourcing location for trials from the West, a domestic-oriented approach would involve partnerships between biopharmaceutical firms and consortia of urban hospitals working with county and township health centers. By building a national clinical trial program, Chinese firms and Chinese physicians would gain expertise in managing multi-sited trials. The country’s potential to emerge as a global leader for clinical trials was downplayed by analysts in the mid-2000s, who instead expected India and Russia to dominate (Bailey 2006). However, India has advanced policies that strongly favor generic drug firms while trials in Russia have proven difficult to manage. As a result, the door is open for China to become a predictable and secure site for large-scale clinical trials of new medicines, devices, and surgical procedures. The key next step involves expansion of pharmaceutical regulation to ensure that human subjects are protected while physicians and sponsor firms adhere to testing norms.

#### Electronic medical records

Electronic medical records (EMRs), offer a second area of biomedical knowledge development in which PPPs can respond to domestic health needs and generate international revenue. In late 2012, the Ministry of Health began to solicit expert input on the basic structure of a system that will include: “summarized medical records, outpatient services, hospitalizations, physical check-ups, referrals, [and] medico-legal statements and reports” (Interfax China 2009). Even as the government develops standards for EMRs, it already has announced a 3-stage plan for their adoption. Initially, hospitals are to process financial, drug prescription, and other basic data through computers. The second stage, termed “information system construction”, involves the storage and management of full clinical information on every patient. The third stage is targeted to information sharing across municipalities and eventually, across the country (Xu et al. 2011; Tu et al. 2012). As of late 2012, over one-third of first and second-tier hospitals were using EMRs, and 96 hospitals across the country were involved in a pilot project to test more complete adoption of e-health systems (Ministry of Health 2012b).

China is especially intriguing for the development of EMRs because it has little legacy of paper patient records held in clinics or physicians’ offices. Instead, Chinese citizens commonly have a small booklet containing records from past doctor visits and hospitalizations. Patients’ ownership and management of their own health records in China fits uneasily with the more centralized systems commonly associated with EMRs. Standards and technologies necessary to EMR implementation are owned and managed by hospitals and IT companies. At present, health data are the property of the government. If China creates an EMR system in the same way as in most Western nations, patients will be shifted to a more passive role relative to physicians and hospitals.

Alternatively, an EMR system that involves increasingly tech-savvy Chinese in the management of their medical data holds significant promise. Key features would need to include access from mobile devices, two-way interactions with medical authorities, and access to data for biomedical researchers domestically and internationally via subscription. In this way, patients could remain involved with their own medical care even as the delivery of care improves through better knowledge about health outcomes. At present, EMRs are built through contracts between public hospitals and private vendors; however, future PPPs between healthcare IT firms and hospitals could foster the creation of data that is also useful to biomedical researchers.

## Conclusion

Whether the product of formal legislative design or a tacit market-based outcome, health systems reflect underlying divisions of responsibility and authority between public and private organizations. Healthcare reform processes expose tensions that are not easily resolved, even with additional spending. China’s healthcare system in the early 2010s faces contradictory mandates of providing baseline coverage to a population of over 1.3 billion, improving the quality of care, deepening the expertise of practitioners, regulating the safety and quality of medicines, promoting innovation of devices, drugs, and surgical techniques, and fostering more systematic TCM approaches. Healthcare also has become an integral part of the government’s plan to achieve the status of a “moderately wealthy” country in the near term. But the relative roles of public and private are still being negotiated for insurance and care delivery, in contrast to other countries where this boundary is more firmly established.

In contemporary China, there is little private insurance coverage and providers operate with low margins. The system instead relies heavily on baseline insurance under government programs and co-insurance by individuals out of their savings. Health economists have suggested that in an optimal co-insurance model, individuals are covered for unexpected or financially catastrophic disease, but do not pressure physicians for excess treatment since they must pay for it directly (Ma and McGuire 1997; Eggleston 2000). In China, however, out-of-pocket spending is extremely high and contributes to a world-leading savings rate. In effect, the Chinese are deferring present consumption out of concern for future medical bills. Proposals for new government-backed catastrophic plans seek to create a broader redistribution system. Under this scenario, China will have public insurance at the top and bottom of the cost scale, and out-of-pocket or private insurance for the middle. However, that development may severely constrain the development of private insurance by making it too difficult for insurers to create profitable patient pools. Catastrophic insurance also may drive up costs as procedures price at or above the coverage baseline.

Future growth of private insurance plans, specialty clinics, and private hospitals in China hinges on the resolution of a complex two-sided dilemma. For insurers to expand and sell price-differentiated plans, clinics need to offer high-quality services covered by the insurance. Public hospitals, which bill patients at government-set prices at the point of service are not equipped to handle post-hoc private insurance reimbursement. But investors need to be convinced of the viability of private insurance plans in order to underwrite new private clinics. An integrated insurance and care delivery model could break through this dilemma only if it simultaneously recruits respected physicians and a broad enough pool of customers that pay annual insurance premiums. If private insurance develops in this manner, it will reduce some of the pressure on the public system but raise tensions concerning inequality of access to top care facilities and providers.

Healthcare systems are organized through core societal compromises about the role of the state and private enterprise. In turn, choices made about the boundary of public and private are revealing of national development goals and strategies. As China plans further reforms, policymakers are grappling with the dilemma that a fully socialized system could deepen coverage, but at the expense of incentives for biomedical research investments. Alternatively, a fully free-market system might better reward physicians and promote innovation, but at the expense of universal insurance and access. Central planners hold a vision of state-run institutions at the center, for-profit firms in supplementary roles, and other non-profit health entities making contributions through nursing homes and end-of-life care (National Development and Reform Commission 2009). The development of more broadly accessible health insurance is a vital next step in China’s transition from a savings and investment economic development model to consumption driven growth.

## Endnotes

^a^Contextual translation of press conference videos posted at: China.com (2012) Press conference of medical and health system leadership team. http://www.china.com.cn/zhibo/zhuanti/2013lianghui/content_28211342.htm. Accessed 29 March 2013.

^b^An exchange rate of 6.25 RMB per 1 USD is used throughout the article, based on the average for calendar year 2012.

^c^This estimate is based on cases compiled at: China Daily Forum (2012).

^d^Author’s analysis of data from P.R. China, *Statistical Yearbook*; OECD iLibrary; ClinicalTrials.gov; and IMS health.

## Author’s information

Arthur Daemmrich is an associate professor at the University of Kansas Medical Center in the Department of History and Philosophy of Medicine with faculty affiliations in the Department of Health Policy and Management and the Department of Preventive Medicine and Public Health. Contact: adaemmrich@kumc.edu
